# Lorna Wing OBE, MD, FRCPsych Formerly psychiatrist and physician, Social Psychiatry Unit, Institute of Psychiatry, King’s College London, co-founder of the UK National Autistic Society

**DOI:** 10.1192/pb.bp.114.048900

**Published:** 2015-02

**Authors:** Christopher Gillberg

**Figure:**
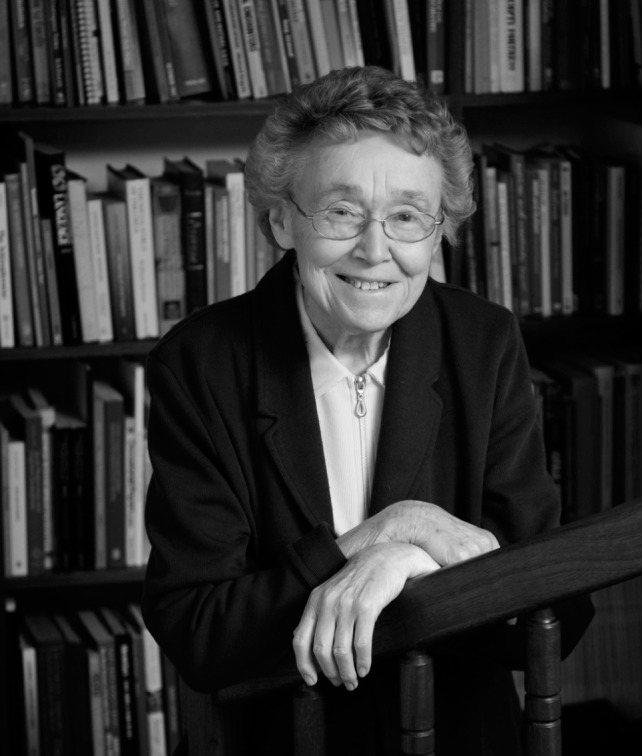
Lorna Wing. © Rex Features.

The psychiatrist and autism researcher Lorna Wing, OBE, has died at the age of 85. Lorna Wing was the figurehead of autism knowledge and the one person in the field of autism research who always had it right, right from the start. She has been a pioneering figure both clinically and scientifically. A whole world including colleagues, patients and relatives mourn her death. She contributed to a better quality of life for millions of people with autism.

Lorna Wing studied medicine at University College Hospital Medical School, London and specialised in general psychiatry shortly after qualification. After obtaining her specialist diploma she was appointed in the mid-1960s to a position in the Social Psychiatry Unit at the Institute of Psychiatry, where she worked until she retired. In the 1960s Lorna, with other parents, was deeply involved in the formation of the UK National Autistic Society, aiming to develop better services for children and older people with autism. The organisation established the first specialist schools for children with autism and later also adult services.

After retirement from university employment, Lorna continued to be professionally active. In 1991, together with Judith Gould, under the auspices of the National Autistic Society, she co-founded the Centre for Social and Communication Disorders for diagnosis, assessment and guidance for people of all ages. In 2008 this was re-named the Lorna Wing Centre for Autism. Lorna Wing specialised in general psychiatry after medical school and worked in the 1960s, 70s and 80s at the Institute of Psychiatry in London, where her landmark studies on social communication disorders changed the concept of autism. Her early epidemiological work together with her husband John and Victor Lotter, and later with Judith Gould resulted in a new delineation of the syndrome, a so-called triad of impairments in the areas of social interaction, social communication and social imagination, which later – in a modified form – became the definition of autism. The three problem areas are still referred to as ‘Wing’s triad’.

In the 1980s Lorna Wing launched the concept of an autism spectrum, and – together with our group in Gothenburg – she was among the first to realise that autism could be considered dimensionally, have very many different aetiologies and affect all age groups and people at all levels of intellectual abilities. When, together, we published reports in the 1990s stating that the likely prevalence of autism was about 1%, other groups considered this to be overstated, but time has supported the claim, and Lorna had it right, right from the start.

In 1981, Lorna Wing launched the term Asperger’s syndrome in a scientific paper in *Psychological Medicine*. She described Hans Asperger’s ‘autistic personality disorder’ and speculated about outcome and aetiology. Thanks to this publication, Hans Asperger’s findings from the 1940s were also introduced to the English-speaking part of the world. Since then, Asperger’s syndrome has become one of the most talked about diagnoses and concepts in clinical medicine.

Based on her vast experience of individuals within the autism spectrum, she outlined different trajectories for development into adulthood: the active-but-odd, passive, aloof and rigid groups. Together with our group she was involved in friendly debates about whether or not all people might actually be reasonably characterised as falling into one of these subgroups and it is only the addition of autism that makes each subgroup stand out much more clearly as a ‘specific type’.

Together with Judith Gould, Lorna Wing developed the most comprehensive autism diagnostic interview in the field, the DISCO (Diagnostic Interview for Social and COmmunication disorders). This instrument is widely used both in clinical settings and research to help in the diagnosis of autism spectrum disorders and related conditions. The DISCO has been translated into several languages and is used all over the world.

Lorna and John Wing had a daughter, Susie, who had severe autism. When Susie died unexpectedly at the age of 49, I remember Lorna’s devastation. She described how Susie had never been able to clearly express her emotions, but how, when Lorna or John came home after work, her face would light up. Lorna said that the feeling that filled her then was absolutely wonderful, unlike anything else.

Lorna Wing published several influential books and about 60 major scientific papers, the last one in 2013, at the age 85. She was the recipient of many honours including the Order of the British Empire.

Lorna Wing also contributed to the development of the recent ‘autism pride’ movement. She believed, and would let people know that she did, that to be really successful in science and arts you need to have some clear autism traits. She also believed that most of us have some such traits. One of her favourite sayings was that ‘nature never draws a line without smudging it; you cannot separate into those ”with” and ”without” traits as they are so scattered’.

Lorna Wing was one of those rare individuals without a false note in her. She was one of the gentlest people, always generous with her time if she believed she could be of help, humble, but never meek. She had a fierce intellect, but she used it in such a way that some of her contemporaries did not understand what hit them until it was too late. She would not suffer pomposity. She had the greatest sense of humour, and hearing her special laugh (and she did laugh a lot) was always a treat and it would put everyone around her in a good mood.

To paraphrase Winston Churchill: ‘Rarely has one person had such an enormous impact on the lives of so many people with autism’. Lorna Wing will live on in many of us who had the great privilege to count her as a true friend.

